# Acceptability of menstrual products interventions for menstrual hygiene management among women and girls in Malawi

**DOI:** 10.1186/s12978-020-01045-z

**Published:** 2020-11-23

**Authors:** Christabel Kambala, Angela Chinangwa, Effie Chipeta, Belen Torondel, Tracy Morse

**Affiliations:** 1 The Polytechnic, Environmental Health Department, University of Malawi, Private Bag 303, Chichiri, Blantyre 3, Malawi; 2 Department of Public Health, School of Public Health and Family Medicine, College of Medicine, University of Malawi, Chimutu Building, Private Bag 360, Chichiri, Blantyre 3, Malawi; 3 The Center of Excellence in Public Health and Herbal Medicine, University of Malawi, Blantyre, Malawi; 4 London School of Hygiene and Tropical Medicine, Keppel Street, London, UK; 5 Centre for Water, Environment, Sustainability and Public Health, Department of Civil and Environmental Engineering, University of Strathclyde, Glasgow, UK

**Keywords:** Menstrual hygiene management, Menstrual products, Acceptability, Malawi

## Abstract

**Background:**

A key challenge for menstruating girls and women in low-resource countries is the inadequate and unreliable supply of menstrual products. Although development partners are implementing menstrual product interventions to address this challenge in Malawi, there is a paucity of information on the distribution of menstrual products and the acceptability of these interventions among users.

**Methods:**

We conducted in-depth interviews with girls (n = 20) and women (n = 26) and 4 focus group discussions (FGDs) with women (n = 35) and 7 FGDs with girls (n = 60) to explore the acceptability of menstrual products interventions in 8 districts. Teachers (n = 12), community leaders (n = 6), community health workers (n = 8) and service providers (n = 9) were also interviewed to explore implementation issues and their views regarding the effect of menstrual products interventions on girls and women. Data were analyzed using content analysis.

**Results:**

Common menstrual products being promoted include locally made reusable pads, commercially made disposable pads and menstrual cups. Overall, adult women preferred reusable pads and young girls preferred disposable pads. Reported benefits of using any type of material distributed included enhanced cleanliness and reduced school absenteeism for girls. While community leaders and teachers applauded the use of disposable menstrual products they expressed concern that they are not affordable for an average Malawian and bemoaned the indiscriminate disposal of used disposable pads. Women and girls highlighted their lack of facilities to effectively wash and dry reusable pads. Service providers bemoaned poor coordination and the lack of national standards to regulate the quality of menstrual products distributed at national level. Improved inclusion of males and health workers could enhance the sustainability of programmes.

**Conclusion:**

While the available menstrual products interventions are acceptable among participants, we note several challenges including affordability, poor disposal methods, lack of attention on sanitation facilities and the lack of standard protocols to regulate the quality of menstrual products. Recommendations to address these issues are reported.

## Plain English abstract

Menstruation hygiene management (MHM) plays a significant role in the reproductive health and well-being of women and adolescent girls. However, menstruating girls and women in low-resource countries have inadequate and unreliable supply of materials to manage their menstruation and resort to using unsafe materials. Development partners are promoting the use of safe menstrual materials to address this challenge in Malawi. We conducted in-depth interviews with girls (n = 20) and women (n = 26) and four focus group discussions (FGDs) with women (n = 35) and seven FGDs with girls (n = 60) to explore acceptability of the menstrual materials in 8 districts. Teachers (n = 12), community leaders (n = 6), community health workers (n = 8) and service providers (n = 9) were also interviewed to hear their views on the same. Data were analyzed using content analysis. The results show that locally made reusable pads and commercially made disposable pads are accepted than menstrual cups. While adult women preferred reusable pads, young girls preferred disposable pads. Perceived benefits of using any type of material included enhanced cleanliness and reduced school absenteeism for girls. Community leaders and teachers applauded the use of the menstrual materials but expressed several challenges including affordability, poor disposal methods, lack of attention on sanitation facilities and the lack of standard protocols to regulate the quality of menstrual products. Recommendations to address these issues are reported.

## Background

Menstrual hygiene management (MHM) is defined as *“Women and adolescent girls using a clean menstrual management material to absorb or collect menstrual blood, that can be changed in privacy as often as necessary for the duration of a menstrual period, using soap and water for washing the body as required, and having access to safe and convenient facilities to dispose of used menstrual management materials”* [1]. Every day, nearly 200 million menstruating girls and women in low- and middle-income countries (LMICs), especially those coming from low economic status households, struggle with MHM [2–7]. They cannot afford to purchase sanitary pads and resort to using unhygienic and improvised menstrual products, which have been associated with urogenital infections [8–10]. When women and girls are privileged enough to have adequate menstrual products, private places to change, especially for adolescent schoolgirls, remain scarce [4]. And when convenient facilities to change do exist, an adequate system to dispose of the menstrual materials (e.g. bins) more than often does not [11–13]. Another challenge is the access to appropriate water, sanitation and hygiene (WASH) facilities, for women and girls to manage their menstruation hygienically, which has also been associated with different reproductive tract infections [8, 9, 14, 15] and with psychosocial stress outcomes [16]. In addition to the direct health implications, poor-quality menstrual products do not effectively absorb and collect blood which leads to menstrual leakage and stained clothes [17–22]. This has psychosocial effects such as embarrassment, shyness, anxiety, shame and stigmatization [4, 23]. To avoid such, girls and women prefer to manage menstruation at home. Consequently, women tend to miss valuable productive work hours and time in public spaces simply because they are menstruating [24], and adolescent schoolgirls often miss school and sometimes end up dropping out of education [25–28]. Ultimately, menstruation has had a significant impact on the social and family life, education, work, and general wellbeing of women and girls [12, 24–28].

Various providers, such as UNICEF, are working with governments and the private sector in LMICs to ensure women and girls have access to a range of affordable and appropriate menstrual hygiene products [1].

In Malawi, literature on MHM is scarce but anecdotal evidence and grey literature suggest that MHM is a challenge for women and especially for adolescent girls in schools and those in rural and/or low socio-economic status households [29]. Apart from inadequate sanitation infrastructure that can accommodate menstruating girls at school, they also have limited access to affordable and hygienic menstrual products and old cloths are commonly used during menstruation [29]. Most girls use a rag from old clothes, as a sanitary cloth, which is folded into a pad and looped about a string around the waist and kept in place with underpants [29]. Girls report several challenges with the menstrual cloths including smelling, leaking and falling out at school [29]. Similar challenges are experienced by older women. Responding to this challenge, a large pool of developmental partners have started distributing menstrual products for the purpose of women and girls upgrading themselves from the “use of old cloths”. These menstrual products, commercially and locally manufactured, are either distributed for free (especially in schools) or sold at a subsidized fee. The products purport to have better soaking abilities to help manage menstruation effectively. However, a substantial knowledge gap remains as to what menstruation products are available nationwide and their acceptance by women and girls is also not yet known. A broader study investigated the status of the menstrual products interventions in Malawi but in this specific paper, we report on the acceptability of the menstrual products among users.

## Methods

### Study design

We used an exploratory qualitative study design where we conducted in-depth interviews (IDIs) and focus group discussions (FGDs) with women and girls to understand their acceptability of the menstrual products distributed. We also conducted key informant interviews (KIIs) with various stakeholders to understand contextual and implementation issues regarding the menstrual products interventions. This study took place from May to October 2018.

### Setting, sampling and data collection

We purposefully sampled a total of 8 districts representing all three regions of Malawi: 3 in the southern (Zomba, Chikwawa, Blantyre), 3 in the central (Lilongwe, Salima, Nkhotakota) and 2 in the northern region (Mzimba and Rumphi). These districts were chosen because they benefitted from menstrual product interventions and the selection of districts in each region ensured that the study captured the cultural diversity prevailing in Malawi. The broader study, which investigated the status of menstrual products interventions in Malawi (forthcoming), compiled a list of all the menstrual products interventions going on in Malawi. We used the list to select the eight districts from all the three regions in Malawi. Based on the list, we used convenience sampling to select schools (n = 8) and communities (n = 8) (1 community and 1 school in each district) for the qualitative interviews. It should be noted that the sites visited were predominantly rural because this is where the majority of the interventions are concentrated. At each selected site we purposively sampled women, girls and other relevant stakeholders for the interviews using the criterion below.

### Inclusion criteria

1. *Women in communities* Female aged 18 and above and still menstruating *or* out of school young females aged 10–17.
2. *Girls in schools* School going adolescent girls aged 10–18; currently enrolled in school and menstruating.
3. *School staff* Involved in teaching and learning in the target school.
4. *Community leaders* Traditional or religious leaders conversant with MHM in target community.
5. *Health workers* Working in a health centre that catered to the study location; conversant with the cultural practices of the study area.
6. *Service providers* Directly involved in implementing MHM programs in the study location
7. All participants had resided in the area for at least 6 months prior to the interview.

Participants were purposefully selected in at least one community and one school in each district. In-depth interviews were conducted face-to-face with 20 girls attending school and 26 women in communities, hence a total of 46 interviews were conducted in the 8 sampled districts. The interviews which lasted between 15 and 30 min were conducted to understand the participants’ acceptability of the menstrual products being distributed and promoted in their schools and communities by development partners.

We also explored acceptability of the menstrual products at group level and so we purposively selected participants in communities and schools to participate in FGDs. These participants were different from those who participated in the IDIs. We conducted a total of 11 FGDs with women/girls (n = 4) in the community and with girls (n = 7) in schools. A total of 60 girls and 35 women participated in the FGDs in the 8 districts that we purposefully sampled. Discussions were designed to obtain full information on the menstrual products provided and explore participants’ acceptability of the products. The FGDs lasted between 30 and 50 min.

KIIs were conducted with relevant stakeholders with the aim of triangulating the IDI and FGD results. The study population for the KIIs included school staff responsible for the girls, health workers covering the study location, service providers providing the menstrual products interventions, and community/traditional leaders conversant with MHM activities. We conducted a total of 35 KIIs with school teachers (n = 12), community leaders (n = 6), community health workers (n = 8) and, MHM service providers (n = 9) also purposefully sampled. The KIIs focused on stakeholder experience of delivering MHM programs, how the programs are being implemented and their views on its effect on girls and women with regards to MHM. We used semi-structured interview guides for IDIs, FGDs and, KIIs.

In the southern and central region, data were collected in Chichewa language and in the northern region data were mainly collected in Tumbuka language. All the interviews and FGDs were audio recorded and later transcribed and translated.

### Data analysis

Data were transcribed, translated, cleaned and then analyzed manually but we used Microsoft Word to organize the quotations. We used content analysis to identify key themes. Preliminary reading of all transcripts was undertaken to get an initial sense of the data and to identify issues for further consideration. We used a direct approach to coding and analysis where specific themes were already arranged as guided by literature [30]. However, the researchers allowed for additional new and relevant themes and subthemes to emerge during the analysis. Similar themes were grouped, and connections and inter-relationships between and within themes were constructed. Triangulation across data sets supported and confirmed the emerging themes.

### Ethical considerations

We obtained ethical approval of the study from College of Medicine Research and Ethics Committee (COMREC) (Protocol number P.01/18/2343). Written permission was also obtained from The Ministry of Education, Science and Technology, The Ministry of Health and The Ministry of Local Government. Prior to the FGDs, KIIs and IDIs, all participants in this study provided written informed consent. For those who were unable to read or write, a thumb print was used. For girls under 18, a written informed assent was obtained from their parents or guardians. All study participants were assured of confidentiality and anonymity. All study data, including interview recordings, and transcripts, are kept in password protected computers and printed documents in lockable file cabinets.

## Results

The findings reflect perceptions of women and girls with regards to acceptability of the menstrual products they were supplied with. Findings also reflect stakeholders’ views regarding the effect of the products on girl’s and women’s day to day lives, and opinions regarding MHM implementation in designated schools and communities.

We found that there were nine MHM service providers working in the eight districts that we sampled. The organizations have both long and short history working in the WASH sector. The service providers that we interviewed in this study were distributing the menstrual products for free. While 1 service provider was solely focusing on distribution of menstrual pads in schools, the other 8 service providers also incorporated MHM activities such as educating users on how to use, dry, store and/or dispose (in case of disposable pads) menstrual products. They further worked with cultural custodians to break taboos around menstruation and reduce misconceptions. Common menstrual products being distributed and promoted in schools and communities included locally made re-usable sanitary pads (n = 4), commercially made disposable sanitary pads (n = 4) and menstrual cups (n = 1). For the re-usable sanitary pads, the organizations train mother groups and standard eight girls on how to sew them. The groups are given sewing machines and materials to make the re-usable pads for their use and the excess can be sold to other girls and women in the communities and surrounding areas to make money in order for them to sustain the venture. The disposable pads are commercially made in the country and the organizations purchase them in bulk for distribution to girls in schools for free. For the menstrual cups, the organization imports the cups from United States of America and distributes to women and college girls for free.

Major and sub-themes that emerged from the data analysis are shown in Table 1.

**Table 1:** Major and sub-themes

Major themes	Sub-themes
Perceived benefits of menstrual products	Reduced absenteeism
	Reduced shame due to staining
	Secretive, one can go unnoticed
	Enhanced cleanliness and reduced odour
	Cultural beliefs dying out/breaking taboos
Ease of use and appropriateness for skin	Disposable pads easy to use
	Disposable pads have enhanced soaking abilities
	Some reusable pads are hard, cause skin irritations
	Menstrual cups complicated to use
	Myths associated with cups
Affordability and accessibility	Disposables expensive
	Reusable pads cheaper and preferred
	Long distances to access materials for making reusable pads
	Shy to ask for pads at school
Disposal and care	Indiscriminate disposal for disposable pads
	Drying places for reusable pads a challenge
Implementation issues	Lack of funds to sustain the programs
	No coordination and poor monitoring
	Lack of standard protocols to regulate quality

### Perceived benefits of menstrual products

Participants at all levels (i.e. IDIs, FGDs and KIIs) reported that they were happy with the introduction of menstrual products interventions (both disposable and reusables) especially in schools. Some service providers felt that before the menstrual products interventions were introduced in schools, there were times girls would drop out of school after staining their uniforms with blood and being laughed at by boys. They appreciate that these programs have also reduced school absenteeism among girls. For example, in the northern region of Malawi, girls would miss school due to cultural practices and lack of materials to use when menstruating as explained by a service provider.

“*Traditionally, when a girl has her first period, she is required to be kept in closed doors for almost 7 days* [also not allowed to go to school]*and also she has to take her bath at the stream or use a separate bathroom from the rest of her family members. After finishing the period, part of her hair is cut, symbolising maturity and readiness for marriage. The girl is regarded as an outcast, she is not allowed to visit the garden as she is cursed and is believed to have the power to curse the plants*”. (KII, female service provider, Mzimba).

Community leaders expressed that issues of menstrual hygiene have improved in their communities. In the past people would notice if a girl was on her period because they would use old and big cloths that would be noticed under one’s clothes.

*“Nowadays, there is no way a parent would know that their child is menstruating as they are now able to manage their menses properly with the pads given”.* (KII, female community leader, Lilongwe).

Teachers were also happy that girls stay clean during the whole day when they are at school.

“*There is a great change on menstrual hygiene management so far. There are no more bad smells in the classes or in girls’ rooms*”[boarding]. (KII, School staff, Blantyre).

Some of the cultural beliefs and practices associated with girls during menstruation such as denying girls talking to male figures like teachers, not cooking and not passing some areas, are dying out due to the presence of MHM interventions in the study areas.

“*In the past, culture dictated that girls are not supposed to be in the same environment with boys or any man during their menses until they finish*”. (KII, School staff, Zomba).

These practices negatively affect the social life of girls and their studies. Organizations are now involved in working with cultural custodians to try and help communities understand that menstrual hygiene is a normal process. An example outcome of this is in one district in the northern region, which has instituted bylaws to ensure that no girl misses school due to menstruation, otherwise parents or guardians are fined. As a result, girls are no longer kept indoors after their first period.

### Ease of use and appropriateness for skin

Most women and girls expressed that they found it easy to use disposable pads when taught how to use them. Some girls felt that the disposable pads were difficult to use. For example, at one school in the northern region, girls ended up peeling off the top layer of the pad (left with a crumbled pad) because their male mentor was not able to explain to them on how these sanitary pads ought to be used. The girls then concluded that the disposable pads are not effective and were unwilling to use them again. Despite this experience, overall, girls expressed that they prefer disposable pads over reusables (both the reusables distributed and the traditional reusables [cloths]).

Preference for the reusable pads with both girls and women, depended on the type of materials that had been used in manufacture. Hard materials, such as cheap cottons, were reported to cause skin irritations and make it hard to walk especially when they become wet.

“*We told them we wanted some soft materials as the vagina is soft and hard cloths may cause sores that might lead to infections*”. (IDI, Community woman, Salima).

However, some girls felt that well-made and designed reusable pads were good for both day and night use as they have a waterproof layer to minimise leakages and a button underneath to keep it in place.

“*You can run or even play netball*”. (IDI, School girl aged 17, Zomba).

Management of reusable pads was also raised as a concern. Girls mentioned challenges with resources, as they required time to wash them with soap, which was not always available. Women also voiced skepticism to use reusable pads because they are not sure if the pieces of cloth they use to make the pads is absolutely clean after washing.

“*Cloth bought from open markets can bring skin rash or other diseases such as candidiasis when used to make reusable pads*”. (IDI, Community woman, Rumphi).

Both groups also indicated reusable pads were not ideal when one is travelling.

While many elements that characterize disposables and reusable pads were appreciated, some women preferred their traditional pieces of cloth (particularly old pieces of blankets), which they reported to have better soaking abilities than the disposable pads. This was specific for someone who is menstruating heavily, and they expressed that the cloth can be folded according to one’s liking and one can use a big piece of cloth to absorb a lot of blood.

Most women and girls felt that menstrual cups were more complicated and challenging for girls to use than sanitary pads. In Rumphi district, menstrual cups were introduced to some female teachers as a pilot. They however complained that the cups were too big and they are only convenient to those with private places to easily change. Some women in Salima district reported to have knowledge regarding menstrual cups for MHM. However, they had never used the cups and were puzzled as to why they should be inserting any foreign object into the vagina, as they have always been advised against the practice by nurses.

“*A nurse told us that we are not supposed to insert anything in there, so why are they now saying that we can be inserting these cups into the vagina*?”. (IDI, Community women, Salima).

Some elderly women think the use of disposable pads removes the “natural flavour” of a woman’s body because of the chemicals used in making them and they also feel that menstrual cups may disfigure their vaginas.

“*We feel that these cups can make our vaginas big and our husbands might leave us*”. (FGD, community women, Rumphi).

“*Menstrual cups block the urine path*”. (FGD, Community women, Rumphi).

2 out of the 9 health workers we interviewed for this study, felt that disposable pads may cause cervical cancer and they felt that the use of pieces of cloth by women and girls when they are menstruating is safe when it comes to contracting diseases or infections.

### Affordability and accessibility

In the case of schools providing disposable pads to students, we found that pads were not given out to every adolescent girl in the schools studied. In some schools, they reported that they had a list of beneficiaries that they help with the pads. In other schools, the disposable pads are given to help the girls manage their first menstruation when it starts at school, but they had to manage the remaining menses on their own. For those who did not benefit from the disposable pads in schools, they indicated that they were hardly available from shops but if available, felt they were sold at quite a high price (75 cents to 1.40 USD per pack of 10 pads). Most of them cannot afford to purchase them every month and therefore resort to using the traditional pieces of old cloth to manage their menstruation.

“*Disposable sanitary pads are very expensive and are scarce in rural area shops*”. (IDI, Community woman, Mzimba).

For both girls and women, they expressed that the availability of money determines the choice of which type of menstrual product to use and reported interchanging disposable pads with old cloths when necessary.

Some girls said they use sanitary pads during the first 2 days of menstruation because of heavy bleeding and then use pieces of old cloth for the remaining days to save on pads.

“*Pads are used when money is available and pieces of cloths or blanket are used when there is no money*”. (FGD, School girl aged 16, Rumphi).

In general, reusable pads and menstrual cups were deemed by the participants as a better option in terms of affordability and disposal. They both require a once off investment, whereby once you buy/receive the reusable pad it can be reused several times depending on the type of material. And the menstrual cup can be used for several years.

Due to the high cost associated with disposable pads, organizations are trying to make reusable pads more available and affordable locally. For example, one organisation donated 204USD to the community women to buy materials such as pieces of new cloth and lining, so that they could make reusable pads for school girls in their area. Girls in this area reported that they prefer the reusable pads because they are given to them for free, but supplies were inadequate to cover every adolescent girl in the community and school.

Despite the willingness of women and girls in the rural areas to support and maintain the availability and production of disposable and reusable pads, some districts (n = 2) highlighted that they have to travel long distances to access pads and materials used to make re-usable pads. In Salima, women indicated that they have to travel to Lilongwe (98.3 km) to buy materials to make re-usable pads, making it expensive and time consuming. Similarly, women in Nkhotakota travel to Salima (111.8 km) to buy disposable pads for their girls as they are unavailable in the local stores.

Compounding the problem for girls is that they are often mocked by boys when they go to buy pads in shops or when they are receiving pads from service providers. This makes girls shy to buy sanitary pads when they find men in the shops. There are also some school girls who are shy to ask for pads from their teachers when they start menstruating at school and pretend to be sick so that they can be sent home.

“*It is very hard at times* [embarrassed to ask] *to let the teacher know that we are menstruating and we feel it is better just to go home*”. (FGD, School girl aged 17, Blantyre).

This was confirmed by another school girl in Nkhotakota who said:

“*When I am shy to tell the teacher that I want pads because I am menstruating, I write a note and give it to a friend to deliver to the teacher”*. (FGD, School girl aged 15, Nkhotakota).

Currently, there is inadequate involvement of male figures in the schools and the community. The girls mentioned that there are some male teachers who mock the girls when they overhear them asking for sanitary pads from female teachers.

“*It is difficult to make male figures understand that it is also their responsibility to help the girl child with issues of menstrual hygiene*”. (KII, Service provider, Chikwawa).

### Disposal and care

Some community leaders complained of the careless disposal of used disposable pads in their communities.

“*You find some girls disposing of used pads in other people’s compounds and dogs tend to pick them up and shred them throughout the community*”. (KII, Female Community leader, Lilongwe).

Some ladies felt that reusable pads are preferable to disposable pads as they do not need to be disposed of.

“*Disposable pads are just disposed of anyhow by the women and the girls in the community and burning of the pads is not good for the environment, burying the pads is also not good because there is plastic in the pads that take a long time to decompose, if disposed in a pit latrine, it get full fast and if disposed in a WC, it might get blocked*”. (FGD participant, Community women, Salima).

However, as much as reusable pads are not disposed of, they need good conditions to effectively wash and dry so as to avoid infections.

“*If they are hanged in a dark place, flies breed on it and when a person uses them they might cause infections such as mauka [candida]*”. (IDI, Community woman, Salima).

But some girls felt that they would rather use the disposable pads and dispose them of in the pit latrine than use and then wash reusable pads. Some girls said they feel nauseated with the smell of blood when washing the used pads. And some girls said using reusable pads is just like using pieces of cloth which is deemed old fashioned. Reusable sanitary pads are mostly worn at home for easy cleaning and washing but when they are at school, disposable pads are used because they are easy to dispose of and do not require a lot of water.

Water is easily accessible in most of the areas this research was conducted and this helps girls to manage their menses properly by keeping clean throughout the day, the girls indicated that they are able to bath 2 to 3 times a day when they are menstruating. But when they use pieces of cloths and reusable pads, a lot of water is needed so when there are water shortages, they tend to use disposable pads. Water and toilets are available in most of the schools we visited and at 3 out of the 8 schools we visited, soap is also provided for washing. However, in most schools, there are no specific rooms where girls can go and change so they have to queue with other learners and some girls end up going to the bush or home to change and when at home, some girls said they use ash to wash the reusable pads. Out of the 8 schools we visited only 1 of these had toilets with designated rooms where girls can change in private when they are menstruating, 2 had just toilet specifically for girls where they can go and change but they had no running water.

## Implementation issues

### Sustainability of MHM menstrual products interventions

Our findings show that, accessibility to both reusable and disposable pads, after the interventions phase out, was impeded by lack of resources to purchase materials. For example, a teacher at a school in Blantyre said that it is difficult for them to ensure the continuous supply of reusable pads to students because the organizations just provide them with the skill of making reusable pads and the rest is up to them. This means that they have to buy sewing materials on their own to continue manufacturing the re-usable sanitary pads and this is often a challenge since both the communities and the schools have no funds to sustain such activities.

In some instances, there were complaints from mother groups (the community groups that are trained by the implementing partners to produce re-usable sanitary pads at community level) that people in the communities do not buy their reusable pads but prefer disposables. As such, what was thought to be an income generating activity (described earlier) is no longer such, hence affecting the sustainability of the projects.

“*When they have money, they would rather buy the disposable pads because they do not need washing*”. (FGD participant, Community women, Salima).

“*Some girls however are lazy and still ask for money to buy disposable sanitary pads*”. (KII, school staff, Blantyre).

In two primary schools in two districts, Standard 8 (final year of primary school) girls were trained on how to make reusable pads. Consequently, when these girls left for secondary school, there were no longer girls that could make pads and the program ended. Similarly, where mothers groups are used for pad manufacture, the regular change in the membership means that new members require training on how to make the pads for the girls which does not happen.

Also affecting sustainability is poor/lack of monitoring. For example, the school projects are monitored when organizations are implementing the MHM programs on quarterly basis but once the intervention ends, there are no proper channels in place on how these programs can be monitored. Community Health Workers such as Health Surveillance Assistants (HSA) who would monitor the projects once they have phased out, are also left out when these programs are being introduced to the schools. Therefore, it is difficult for them to incorporate MHM into their routine WASH activities when working in their catchment areas. None of the five health workers we interviewed in five districts during this study were included in the menstrual product interventions.

“*There are 14 HSA’s in this area but no single HSA has ever been involved when NGOs are implementing these programs*”. (KII with HSA, Baptist Health Centre, Salima).

There is also a lack of live data regarding who is doing what and where and there is no coordination among the organizations that are implementing MHM programs.

“*When starting these programs there is no information on who does what and where are they*”. (KII, Service provider, Lilongwe).

### Quality of the products

Service providers also expressed that there are no regulations regarding the quality and standard of the menstrual products that they are promoting in the schools and communities. Others thought it would be good to have guidance and/or national standards to regulate quality since some products may be unsafe for use and may be a potential source of infection.

*“As an organisation, we would love the government to prioritise provision of high quality standard sanitary pads and facilities for adolescent girls and finances for the procurement of pads for the learners in the budgets. There are still schools that do not provide places for girls to change when they are menstruating and that there are still girls missing classes because of lack of menstrual products.”* (KII, service provider, Lilongwe).

*“We would also like government to put standards and policies in place that when they are implementing MHM programs, they should also be taking into account quality of pads.”* (KII, Service provider, Salima).

## Discussion

In this study, we set out to explore the acceptability of the menstrual products that are being promoted in Malawi by different developmental partners or service providers. There were nine service providers in the areas where we conducted this study. We found three types of menstrual products which were commonly promoted in the study area: disposable pads, reusable pads and menstrual cups.

The use of disposable pads was favoured by most girls that participated in our study. The girls found this an easier option because one does not require a lot of water to take care of the napkin, and that soaking abilities are enhanced. This is only rational considering that most homes and schools in LMICs (including our study areas) have poor access to clean water, sanitation and hygiene facilities for the girls to manage their menstruation hygienically [31]. However, the cost of the disposable pads was deemed to be high by the standards of most participants. This is understandable considering that an average Malawian lives below $0.50 per day [32] and the disposables cost between $0.75–$1.40 per pack of 10 pads, as such in this context, the use of disposables though preferred may be a challenge. Consequently, as noted in earlier studies, rural and poor women and girls resort to using old cloths which may place them at risk of leaks and potential infections [17, 18, 22, 24, 33, 34]. This calls for free and/or heavily subsidized menstrual products interventions. However, it should also be noted that preference of menstrual products is not only based on economic status but also on personal choice, cultural acceptability, and availability on the local market [11].

Unlike girls, women appreciated the importance of reusable pads more than the disposable pads. They felt they had upgraded themselves from using ‘old cloths’ (usually dirty), which is positive given the fact that the ‘old cloths’ are associated with urogenital infections [8, 9]. However, the study revealed that women from different rural districts of Malawi were sourcing materials from shops in major cities, to which they have to travel long distances. They indicated that the materials which are good for reusable pads are not found locally in their rural areas/districts; the materials they find locally lead to the development of rashes. With such experiences, others preferred their old cloths or pieces of old blanket. Women should be made to understand that there is nothing wrong with using their ‘old cloths’, what matters is the hygiene associated with caring for such materials. These include washing the cloths effectively with water and soap, and finding a suitable place for sun-drying the washed materials. But culturally, in Malawi, it is a taboo for the old cloths to be seen by other people, and as such they are not hung in open spaces. As a result, women and girls end up hanging them in unhygienic conditions that are prone to multiplication of microbiological hazards. Eventually, the use of reusable materials whether commercial or their traditional ‘old cloths’, can still lead to urogenital infections. The importance of hygiene related practices during menstruation can never be over emphasized as it prevents vulnerability to urogenital infections [8–10]. This calls for a comprehensive hygiene education as already pointed out by other studies [9, 19, 35–37].

As shown in the results, reusable pads and menstrual cups were deemed by the participants as a better option in terms of affordability and disposal. However, there are two issues of concern: first, they require high level of hygiene because when changing you need sufficient water to wash the cloth and cup before reusing. This is somehow compromised in populations that are struggling to find water or do not have water in the toilet area when changing especially when women and girls are in public places such as school [2, 3, 12, 35]. If not careful, then it could also be a source of infection. There are also misconceptions regarding the use of menstrual cups. Most women and girls feel the cup can break the hymen, widen the vagina, and subsequently cause dissatisfaction when it comes to sexual intercourse. These misconceptions are consistent with existing literature [38], but they need to be dispelled as myths. More research is needed on this aspect. There is also need to widen the women’s choice by introducing safer and environmentally friendly pads such as bamboo fibre pads, banana fibre pads, and water hyacinth pads which are cost effective, easily biodegradable and ecofriendly [35].

Another key finding relates to poor disposal of pads. As women and girls shift from the use of reusable old cloth to the use of disposable pads, we face a challenge of ensuring safe and adequate disposal methods. Therefore, there is need for proper sanitation and disposal infrastructure to support the safe disposal of these materials without affecting the environment through land (by burying) and air pollution (by open burning) [11, 37]. Available local sanitation masons in WASH sector can be brought in to design safe disposal methods.

As the findings indicate, participants in the study felt that the availability of sanitary products is helpful in making girls stay in school. Community leaders are happy that school dropout rates in their communities have reduced since the introduction of menstrual products interventions in Malawi. Participants in our study also expressed that with the menstrual products, shame due to staining of clothes has reduced, and one can go unnoticed (secretive), enhanced cleanliness and reduced odor. While these findings are positive, there is need for more robust and large scale trials to confirm whether the menstrual products reduce school absenteeism and drop-out rates. As pointed out by Hennegan and Montgomery [13], research is needed to establish the role of MHM in education performance, employment and other psychosocial outcomes [39]. To this end, the authors acknowledge that it is not only interventions that focus on distribution of menstrual products which can reduce school absenteeism, but also multiple interventions which aim to improve WASH facilities, reduce taboos, improve school environment, improve menstrual hygiene, knowledge and practices [40].

One important finding, is that the presence of the menstrual products programs in the areas is supporting the removal of some harmful cultural practices, in that people have started to break the silence on the topic of menstruation, and slowly reduce the fear that was caused by cultural myths. For example, service providers work with cultural custodians to abolish some of the practices that prevent girls from attending school when they are menstruating. This is a move in the right direction, given that the topic of menstruation is still treated with silence and that cultural practices and taboos around menstruation continue to negatively affecting the lives of women and girls in LMICs [4, 5, 41–43]. Negative practices such as restrictions on cooking, working activities, sexual intercourse, bathing, worshipping and eating certain foods [44] and the unsupportive attitudes of some men including teachers as shown in our study, are retrogressive and have negative impacts on the development of girls and women alike. As LMICs are developing, menstrual products are helping women and adolescent girls to manage menstruation hygienically and with dignity. As a consequence, this also supports gender equality, as females can complete their education, and subsequently contribute to developmental activities both domestically and nationally.

As the findings indicate, organizations that are implementing the menstrual products interventions are not able to reach each and every girl and woman in rural Malawi. Those that have benefitted have not been provided with a long term supply, particularly in the case of disposable pads. Mother groups that have been provided with starter pack resources to locally make the pads are also struggling to sustain the projects, as the pads being sold are inadequate to generate money for these programs to be sustained. As Malawi has 50.7% of the population living below the poverty line and 25% living in extreme poverty [32], it is very hard for them to sustain these programs independently. This impacts negatively on the success of the menstrual product interventions as women will not be able to make the pads. Those that buy pads from shops also expressed their concern on the high price of the commercial sanitary pads. This defines more clearly the importance of understanding context when implementing interventions. The management of menstruation has become a social condition that requires understanding and the development of context appropriate interventions to enable every woman and girl to access hygienic menstrual products to absorb or collect blood.

This study also indicates that when organizations are introducing some interventions in the communities, there are some crucial stakeholders which have been excluded, and this makes it hard for the programs to be sustained, supported and monitored when the organizations leave. Inclusion of other stakeholders such as community health workers, parents and community leaders is vital for the continuity of these programs, as they would be able to monitor if the interventions are bringing out the expected results long after the project closes. The lack of standard protocols to guide the implementation of MHM in Malawi is a cause for concern given the many organizations promoting menstrual products in the country. Our study shows that several NGOs, government agencies and the private sector are distributing, or selling at a subsidized fee, menstrual products in at least two or more districts. Given the presence of these organizations across the country, the challenge of assuring high-quality products is even greater. The success of MHM will be led by practical and robust MHM policies, programmes, services and implementation strategies. These will provide a road-map for operations and also ensure that MHM is regulated in Malawi. In addition, the policies will give guidance for decision making, streamlining processes and establishing boundaries for acceptable menstrual products. And implementers should actively involve men so they can be supportive towards menstruating women and also understand the ill effects of poor MHM. As noted earlier, if we want to improve MHM as per the UNICEF and WHO definition, government needs to invest in different aspects including the improvement of WASH facilities, reduction of taboos, improvement of school environment, improvement of menstrual hygiene, knowledge and practices.

## Limitations

The use of non-probability sampling when selecting districts and participants may be one of the weaknesses of our study. However, this was due to the lack of live database of organizations that are on the ground. The study results may not reflect the situation of menstrual products in the urban settings because data collection was predominantly done in rural areas and among those from low income households. Services may differ in the urban context from those in rural settings. Urban settings may have accessibility to more types of products than the three that were identified in this study and women and girls in the urban settings may have a wider choice of what to use for their menstrual flow. As such, acceptability results may not be generalized to the whole country. Further, we did not assess girls and boys knowledge of menstruation and MHM due to time and resource limitations. We also did not interview boys to get their perspectives regarding the menstrual products because we were more focused on understanding the acceptability of the products from the users (i.e. the girls and women). Nevertheless, our results provide useful insights of acceptability of the current menstrual product landscape in Malawi.

## Conclusion

This study has established the baseline situation of the acceptability of menstrual product interventions in Malawi. The results suggest that adult women prefer commercial reusable pads and younger girls prefer disposable menstrual products compared to menstrual cups because of the benefits they bring to both their physical and psychological well-being. Despite the benefits, the study has revealed that affordability, accessibility, disposal and care of used products and management of interventions are continuing challenges that need to be addressed. Specifically, it has highlighted the lack of standards to regulate the quality of menstrual products that have proliferated in the country. Much as these products may be produced and distributed in good faith, some may be unsafe for use and may be a source of infection and detrimental to health, the very thing that we are trying to prevent. We need to examine broader dimensions on how these products should be regulated. Future interventions need to consider these issues when designing and implementing menstrual hygiene programs. There is also need for further exploration of barriers that prevent women from using other menstrual products interventions such as the menstrual cup.

## Data Availability

Data sets are available from the corresponding author upon reasonable request.

## Abbreviations

COMREC: College of Medicine Research and Ethics Committee
FGDs: Focus group discussions
IDI’s: In-depth interviews
KIIs: Key informant interviews
LMIC’s: Low and middle income countries
MHM: Menstrual hygiene management
SHARE: Sanitation and Hygiene Applied Research for Equity
WASH: Water, sanitation and hygiene
WASHTED: Water, Sanitation, Health & Appropriate and Technology Development
